# Schizophrenia and Risk of Dementia: A Literature Review.

**DOI:** 10.1192/j.eurpsy.2024.1583

**Published:** 2024-08-27

**Authors:** V. Moraiti, G. N. Porfyri

**Affiliations:** ^1^Early Intervention in Psychosis, Ikelos NGO, Athens; ^2^Psychiatric Department, General Hospital of Papanikolaou, Thessaloniki, Greece

## Abstract

**Introduction:**

Dementia is a clinical syndrome affecting 1-2% of the population under the age of 65, while at older ages the frequency doubles every five years. The clinical manifestations include memory loss, communication deficits, agnosia, apraxia and executive dysfunction.Schizophrenia is a complex, chronic mental disorder affecting approximately 1% of the population, presenting with disturbances in perception, thought and behavior.

**Objectives:**

To investigate the relationship between schizophrenia and later-onset dementia; more specifically to explore whether schizophrenia increases the dementia risk.

**Methods:**

A review of 35 articles -from 2010 to 2023- on PubMed and Google Scholar regarding patients with schizophrenia or other type of psychosis, who later presented dementia.

**Results:**

Patients with a history of schizophrenia, schizotypal disorder, or delusional disorder are more likely to develop dementia.The greatest risk is presented in patients showing the shortest duration of psychotic symptoms (5 years or less), while at 5-10 years the probability of developing dementia decreases.The most common types of dementia occurring in psychotic patients are alzheimer’s disease (50-70%),vascular dementia (30%) and unspecified dementia (15%).Chronic patients (10+ years of symptomatology) are less likely to develop dementia. Psychotic patients over the age of 65 are more likely to develop dementia later in life, while individuals who develop schizophrenia after their 40s are three to four times more likely to present dementia compared to patients carrying a schizophrenia diagnosis before their 40s.Females with Late-Onset Schizophrenia have an increased dementia risk compared to males carrying the same diagnosis and compared to healthy females of the same age. Physical conditions implicated in the onset of dementia in schizophrenic patients include cardiovascular diseases, lung disease, substance and alcohol use, head injuries and diabetes.

**Conclusions:**

According to data, there is a strong correlation between schizophrenia and dementia.However, the related studies are limited in number, while their results require further investigation because of limitations (small sample sizes, co-morbidities, selection of chronic elderly patients).Furthermore, most of these studies were conducted in Western countries, highlighting the necessity of pursuing additional research.

**Disclosure of Interest:**

None Declared

